# lnc RNAs, hypoxia and metastasis

**DOI:** 10.18632/oncoscience.247

**Published:** 2015-09-23

**Authors:** Benilde Jiménez, María Tiana, Luis del Peso

**Affiliations:** Department of Biochemistry, Universidad Autónoma de Madrid and Instituto de Investigaciones Biomédicas Alberto Sols, CSIC-UAM, Madrid, Spain

**Keywords:** lncRNA, hypoxia, metastasis, HIF, EFNA3

In a variety of pathophysiological conditions oxygen demand exceeds its supply, leading to a compromising condition referred to as hypoxia, which triggers complex adaptive mechanisms mediated primarily through transcriptional reprogramming by hypoxia inducible factors (HIFs). Solid tumors are an example of this condition, as deregulated proliferation generates oxygen deficient microenvironments to which cancer cells need to adapt. Among the adaptive processes, the growth of new vessels (angiogenesis) and metabolic reprogramming are central to cope with low oxygen tension. However, in the context of solid tumors, hypoxic areas perpetuate and expand due to the aberrant activation of angiogenesis which gives rise to abnormal tumor vessels and a dysfunctional tumor vasculature. In fact, the prognostic significance of hypoxic regions is well recognized and tumor hypoxia is causally associated with resistance to therapy. Additionally, hypoxia induces metastasis through regulation of several critical processes implicated in the complex metastatic cascade, especially epithelial-mesenchymal transition and cancer stem cell renewal [1]. Recent studies have implicated HIF target genes in nearly every step of the metastatic process; however, the precise molecular and cellular mechanisms underlying hypoxia-induced metastatic dissemination are just beginning to be uncovered.

In recent years, the genome-wide identification of hypoxia-inducible biding sites and target genes has allowed a more complete understanding of the adaptive response to hypoxia. Using an *in silico* global approach we identified Ephrin-A3 as a potential novel HIF target gene [2]. Ephrin family members mediate attractive and repulsive interactions between cells through signaling by members of the Eph receptor family and function as guidance molecules during neurogenesis and neovascularization. Importantly, some Ephrin family members have been implicated in the promotion of metastatic dissemination. In our recent study published in Oncogene journal [3], we describe that EFNA3 RNA and protein accumulate in response to hypoxia in a HIF-dependent manner and found elevated EFNA3 expression levels in Renal Cell Carcinoma (RCC), as a prototypic cancer in which HIF is stabilized due to loss of function mutations in VHL. Furthermore, we found a positive correlation between EFNA3 expression and risk of metastasis in breast cancer patients. This correlation points to the clinical relevance of the regulatory connection between HIF, EFNA3 and metastatic dissemination demonstrated by our study. As for the mechanism by which Ephrin A3 protein promotes a metastatic behavior, using a combination of breast cancer orthotopic xenotransplantation and co-culture models, we demonstrated that by imposing repulsive interactions between tumor and endothelial cells Ephrin-A3 enhances the ability of tumor cells to intravasate/extravasate into the tumor microvasculature (Figure).

**Figure 1:**
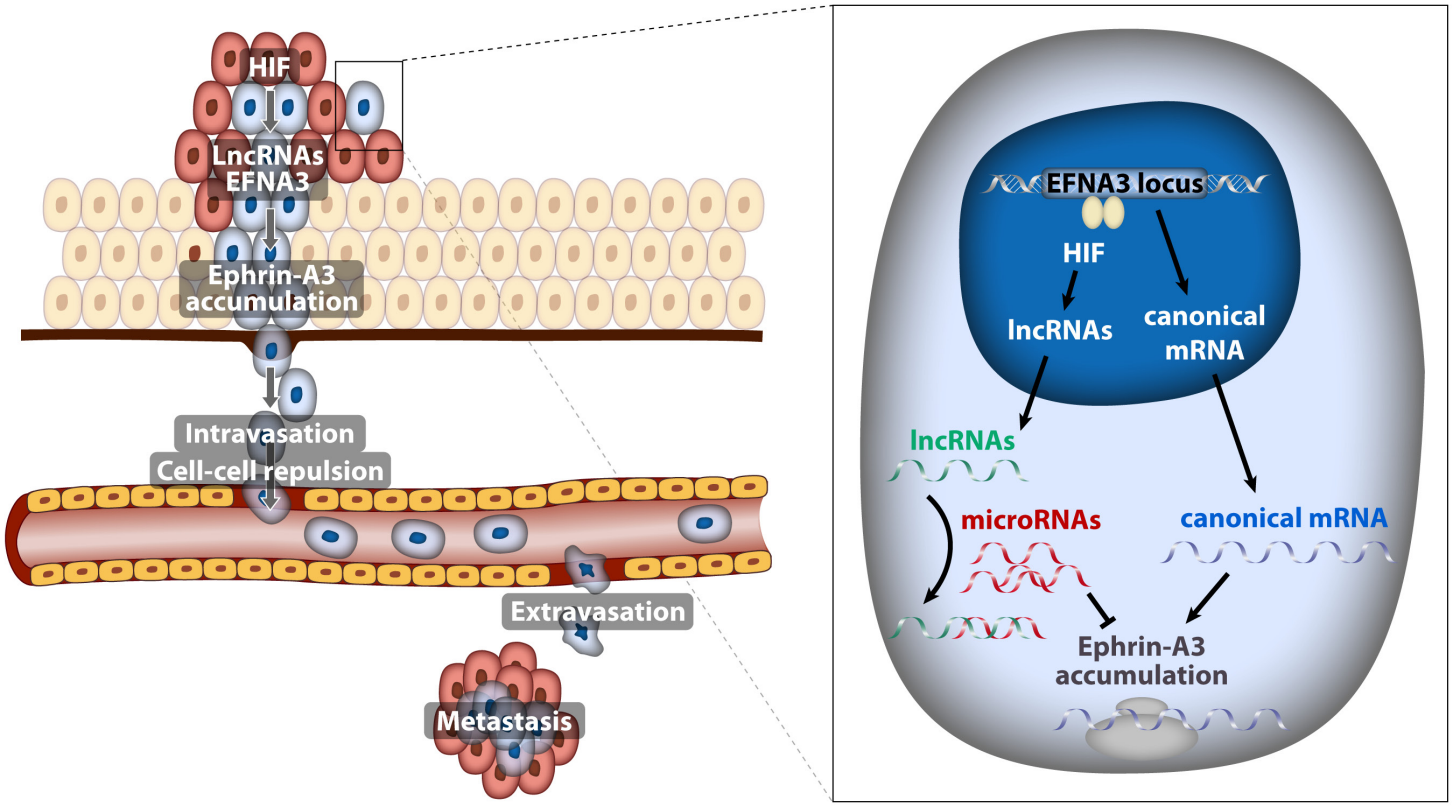
Model depicting the implication of EFNA3 lncRNAs induced by hypoxia in the metastatic dissemination of breast cancer cells

All these experiments suggested a linear pathway in which hypoxia contributes to metastatic dissemination through the HIF-mediated induction of *EFNA3* mRNA and Ephrin-A3 protein accumulation. However, careful examination of the EFNA3 locus revealed that, in addition to the canonical EFNA3 mRNA, it encoded for two long non-coding RNAs (lncRNA) [3] that were not previously annotated and that transcribed from alternative transcription start sites (TSS). This prompted us to investigate which of these transcripts were regulated by oxygen. Unexpectedly we found that, in cancer cell lines, hypoxia led to the HIF-dependent transcription of the lncRNAs encoded by the EFNA3 locus and had only a minor effect on EFNA3 mRNA levels. Moreover, we identified HIF binding sites in the close proximity of the lncRNAs TSS, but not in the region where the canonical EFNA3 mRNA transcribes from. Additionally, VHL deletion in conditional VHL-knockout mice resulted in increased expression of the EFNA3 lncRNAs without significant alteration of the coding EFNA3 mRNA; confirming that *in vivo* EFNA3 lncRNAs are prevalently induced by HIF. These results suggest the apparent paradox of hypoxia leading to Ephrin-A3 protein accumulation through the induction of non-coding transcripts instead of its coding mRNA.

LncRNAs have been implicated in almost every step of gene expression regulation through their capability to bind RNA, DNA or proteins [4]. Thus, we next investigated the mechanism by which the novel lncRNAs impinge on the *EFNA3* locus. By overexpressing the various NC isoforms in breast cancer cell lines we were able to demonstrate that *EFNA3* lncRNAs caused Ephrin A3 protein accumulation. Subsequently we found that *EFNA3* lncRNAs do not affect Ephrin A3 protein stability but rather relieve the canonical *EFNA3* mRNA from the inhibitory action of specific miRNAs allowing for efficient translation (Figure). This novel mechanism suggests a prominent, unanticipated, role of non-coding RNAs in the control of gene expression in response to hypoxia and illustrates the complex interplay among the different classes of non-coding RNAs, including long non-coding and micro RNAs. In this regard, it is worth noting that it has been recently reported that HIF is a major regulator of the non-coding transcriptome [5]. Conversely, some lncRNAs, such as LET [6], regulate HIF stabilization, adding an additional layer of complexity to the role of lncRNAs in the transcriptional response to hypoxia.

In summary, we uncovered a novel mechanism where Ephrin-A3 expression is driven by hypoxia-induced EFNA3 lncRNAs to promote metastatic dissemination (Figure). Recently, other HIF-regulated lncRNAs like lncRNA-AK058003 [7] and UCA1 [8] have been also implicated in the control of the invasive potential of cancer cells. Therefore, identification of the functional roles of the complete set of non-coding transcripts regulated by changes in oxygen tension is of utmost relevance for a deeper understanding of the complex role of hypoxia in metastasis.
